# Wild chimpanzee termite mound inspections converge with the onset of rain

**DOI:** 10.1038/s41598-025-90382-9

**Published:** 2025-04-17

**Authors:** Seth Phillips, Payton Sime, Cielo De La Rosa, Julia Whelan, Jay Reti, Alex K. Piel, Fiona Stewart, Vicky M. Oelze

**Affiliations:** 1 Anthropology Department, University of California at Santa Cruz, Santa Cruz, USA; 2 Department of Anthropology, University College London, London, UK; 3 GMERC, LTD., Mpanda, Tanzania

**Keywords:** Tanzania, Great ape, Tool-use, Weather, Insectivory, Camera-trapping

## Abstract

How and when to inspect for hidden and ephemeral food sources presents a cognitive challenge for wild animals. Observational data suggests a high degree of seasonality in the chimpanzees’ preferred termite prey at the site of Issa Valley, Tanzania. This allows us to consider hypotheses on chimpanzees’ ability to predict termite activity and to efficiently forage for termites based on seasonal rainfall trends. We analyzed 6806 min (113 h) of camera trap videos recorded over 48 months (from 2016–2019) from termite mounds and documented chimpanzee inspection behavior in the Issa Valley, western Tanzania. Our results suggest that Issa chimpanzees initiated foraging for termites, adjusted the period in which they prepared tools in advance, and inspected termite mounds in association with rainfall trends. Chimpanzee planning and foraging behaviors have implications for hominin decision making, forethought, and seasonal landscape utilization.

**Supplementary Information:**

The online version contains supplementary material available at 10.1038/s41598-025-90382-9.

## Introduction

Many chimpanzee (*Pan troglodytes*) communities across Africa are known to make organic tools, insert them into the flight holes of mound building termite colonies, and then extract and consume the termites that latch onto the tool^1,2^. This behavior was first described by Jane Goodall in free-ranging chimpanzees (*P.t.schweinfurthii*) in Tanzania and revolutionized our perspective on tool-use traditions in non-human animals^1^. The specific techniques of this termite-fishing behavior can vary considerably between chimpanzee communities that practice it, and as such termite-fishing is considered a socially learned, cultural behavior^3,4^. Studies on the mental processes necessary to produce and use various tool sets such as those used for termite-fishing have uncovered cognitive abilities of wild chimpanzees^5,6^ in addition to enriching our understanding of great ape culture^7^. For instance, some central African chimpanzees (*P. t. troglodytes*) demonstrate hierarchical organization^8^, or the utilization of a logical behavioral sequence with various steps and subroutines, in their use of 2–3 distinct tools to procure underground termite prey^5,9^.Complex perishable tool use such as this also has behavioral implications for early hominin behaviors and foraging opportunities^10,11^.

Another important aspect to termite-fishing has received far less attention and may offer additional insight into wild chimpanzee foraging cognition: when and how do chimpanzees decide the appropriate times to termite-fish? McGrew and colleagues^12^ found that the frequency of termite-fishing tools discovered a top of termite mounds at Mt. Assirik in Senegal and Gombe in Tanzania increased during their respective wet seasons. Bogart and Pruetz^13^ describe termite-fishing as a year-round staple for Fongoli chimpanzees while also noting termite foraging peaks during the late dry—early wet season transition. However, rainfall was not correlated to total termite-fishing time or bout length for the Fongoli community^13^. Koops and colleagues^14^ found that predation on army ants (*Dorylus* spp.) by the Seringbara chimpanzee population was not related to rainfall though they did find it was associated with availability and altitude. In the Issa Valley of Tanzania, human experimenters have demonstrated that *Macrotermes* are inaccessible to chimpanzee style termite-fishing techniques throughout most of the year^15^. Termites are vulnerable to termite-fishing almost exclusively during the early onset of the wet season. During this time, the colony’s worker caste construct passageways connecting chambers in the nest interior to the mound’s surface for the reproductive alate caste to disperse^16,17^. Following these mound dispersal events, which typically occur before 500 mm of rainfall^15^, successful extraction of termites using chimpanzee termite-fishing techniques was rare for human experimenters. *Macrotermes* are considered a high-value^2,18,19^, ephemeral resource rather than a fallback food for the Issa community^15,20^. Issa chimpanzees would greatly benefit from an ability to predict the availability of *Macrotermes* and adjust their time invested in exploring this valuable resource. The seasonal variability in the availability of termites reported in the Issa Valley allows us to test whether rainfall trends that predict termite availability similarly predict chimpanzee termite foraging behavior.

Field-based observational studies allow researchers to test primate cognitive abilities within a relevant naturalistic context^21,22^. Janmaat^21^ asks three questions that help to establish guidelines for observational studies of wild cognition: “What do chimpanzees fail to find?”, “What do chimpanzees not do?” and “Under what particular conditions do chimpanzees plan?”^21^. In her review, Janmaat outlined examples from her own work in Taï National Park (Ivory Coast) in which her and her team recorded instances of chimpanzees inspecting feeding trees that did not contain ripe fruit^23^, chimpanzees approaching but not inspecting trees^24^, and chimpanzees departing earlier from nests to travel to and feed on ephemeral fruits that may be subject to high competition for feeding^25^. She identified how intuitive statistics, inter-season memory, and planning are necessary skills for chimpanzees to effectively forage on highly ephemeral fruit in their natural habitat^21^. With this framework, much can be learned from the non-events (i.e. bypassing a mound) of termite-fishing, the unsuccessful or “negative-bouts”^26^, as well as from the conditions in which chimpanzees do prepare tools in advance, attempt, and succeed in extracting termites. Studying wild chimpanzee termite-fishing and inspection behavior offers a chance to contextualize observed cognitive skills within a relevant ecological context. Implications of such helps us identify potential adaptive pressures, such as temporality of embedded high-value foods, that may help drive the evolution of intelligence in primates^21,27^.

To termite-fish at a *Macrotermes* mound*,* one must invest time into both tool production and inspection behavior. Teleki^28^ wondered how Gombe chimpanzees in Tanzania were able to even locate flight hole passageways on the mound’s surface. In the Issa Valley, the outlines of *Macrotermes* alate flight holes, which are often covered by a thin layer of freshly worked soil, are commonly visible to human observers. Chimpanzees have been reported to employ a suite of sensory skills to locate productive termite mounds including visually scanning the mound surface^28^, scratching away at freshly worked soil to expose passageways^1^, and sniffing tools to detect termite pheromones after inserting it into the mound^6^. Additionally, constructing tools to termite-fish is a time-costly activity and would not be expected to occur in high frequency unless a reward is anticipated. For example, Musgrave and colleagues^29^ found that chimpanzee mothers that relinquished their tools to offspring spent 33% less time termite-fishing in the immediate 30 s interval afterward. The authors interpreted this reduction in termite-fishing time as a cost to the tool-donor^29^. Tool preparation requires a degree of forethought that is regarded as more challenging, and more rare in the animal kingdom, than just using tools^6^. The majority of plants used to construct tools by chimpanzees in the Issa Valley are located more than 10 m away, and out of sight, from the nearest termite mound^30^. Temporal differences in chimpanzee inspection effort at mounds and tool preparation can help us to elucidate when chimpanzees expect to find termite prey. Table 1 lists the null hypotheses that were tested in this study.

**Table 1:** List of null hypotheses to be tested against mixed-effect models.

Model #	Null hypotheses
1	Chimpanzees arrive with tools regardless of rainfall
2A	Chimpanzees inspect mounds regardless of rainfall
2B	Duration of inspections does not change with rainfall
3A	Termite-fishing success is not influenced by rainfall
3B	Duration of termite-fishing bouts does not change with rainfall
3C	Termite-fishing efficiency does not change with rainfall

In the current study we sought to determine:

1. Whether rainfall predicts a chimpanzee presence and inspection behaviors on termite mounds
2. Any effect of rainfall on the success of termite-fishing for Issa chimpanzees

## Methods

The Issa Valley study area covers ca. 85 km^2^ and is located in within the Tongwe West Forest Reserve in western Tanzania. It is a savanna mosaic habitat with varied vegetation types dominated by miombo woodlands and including grasslands, and thin strips of riparian forests. There are two distinct seasons: the wet season, which typically occurs between November and April when nearly all annual rain falls, and a dry season (< 100 mm/month) occurring between May and October. Annual rainfall at the Issa Valley averages around 1205 mm from 2010–2020 (range 907–1748 per annum). The Issa chimpanzee community is known to termite-fish at the large, epigeal *Macrotermes* mounds that are found throughout the different vegetation types^31^. So far, *Macrotermes subhyalinus* is the only species of *Macrotermes* that has been taxonomically identified^32^, including several colonies described in this study.

At 13 *Macrotermes* mounds, we deployed motion-triggered cameras (Bushnell Trophy Cam HD Aggressor, Bushnell CORE) to continuously monitor termite and chimpanzee behavior (for termite behavior see Phillips et al. 2023). Seven of these 13 cameras have been deployed since 2016. We deployed the additional six cameras in October 2018. We placed cameras on nearby trees approximately one meter from the ground, facing the mounds, and set them to record for 60 s intervals when triggered by motion, 24 h a day^15,33^. Every two weeks, we replaced camera batteries and SD cards and downloaded the video data. Between 2016–2019, we recorded local precipitation data every five minutes using an environmental weather logger (HOBO RX3000 Remote Monitoring Station) located at the research station. Due to equipment malfunction, there was a gap in rainfall data from January–April 2017. This gap resulted in 2 observations being excluded from the models that test the effects of rainfall parameters on chimpanzee behavior.

For this study, we first filtered all resulting videos (n = 42,384) to examine only those that include chimpanzees (n = 9,955), which resulted in 6,806 min (113 h) of video footage for analysis. Much of this footage contained more than one chimpanzee termite-fishing simultaneously so the total focal behavioral data is longer than 113 h. Note that sometimes a camera’s settings would reset and have incorrect settings for weeks in which they did not record full 60 s videos but instead recorded 5 or 15 s videos. We organized sequential videos of chimpanzee parties into distinct bouts, based on date, time, and location in which they occurred. Bouts occurring at the same location and day would be separated and classified into new bouts if there was 15 or more minutes in between videos of a chimpanzee at the same mound to try and account for the possibility that one chimpanzee party may leave before another party arrives and begins a separate inspection process within the same hour. An observation (n = 2,006) covers all the behaviors of an individual chimpanzee during a single bout or instances of an individual bypassing a mound. For example, a single observation could include anytime a chimpanzee passes by a mound without stopping. As an alternative example, an observation could be when an individual chimpanzee arrives to a mound with or without tools, inspects the mound, and then attempts to termite-fish.

We constructed our ethogram (Table 2) and used the free open-source Behavioral Observation Research Interactive Software (BORIS) to code behaviors into our ethogram, review footage of chimpanzees at termite mounds, and carefully log events of chimpanzee termite mound inspection behavior and successful termite-fishing for data analysis^34^. Chimpanzee termite mound inspection behavior includes anytime a chimpanzee utilizes their visual, olfactory, or tactile senses to attempt to locate a productive flight hole at a termite mound. Each behavior recorded was associated with a focal subject. We used behaviors of fully weaned individuals (older than approximately 5 years of age) for this study. Chimpanzees were not identified to the individual level for this study. In many instances, an individual’s behavior could not be positively confirmed during portions of the observation because only a portion of their body was visible. We removed all instances in which chimpanzees were at a mound but their exact behavior could not be determined for models testing specific termite-fishing or inspection behaviors.

**Table 2:** Ethogram of chimpanzee behaviors at *Macrotermes* mounds analyzed in BORIS.

Behavior	Type	Description
Fishing duration	State event	Begins after first confirmed successful dip. Ends when an individual leaves a mound or stops termite-fishing
Inspection duration	State event	Begins when an individual employs first inspection behavior. Ends after first successful dip or individual leaves
Presence at mound duration	State event	Begins when individual arrives within approximately one meter of mound. Ends when individual leaves frame for last time
Resting at mound duration	State event	When an individual is at a mound but is not fishing or inspecting for a minute or more
Arrival on mound with tool	Point event	Individual arrives at mound for the first time for that bout with tool already in mouth or hand
Changing insertion in mound	Point event	When an individual changes the flight hole that they are inserting the tool into
New tool	Point event	Individual creates or replaces a new tool (different than arriving on mound with tool)
Olfactory inspection	Point event	When an individual sniffs a tool after inserting it into the mound, sniffs their finger after touching flight hole, or sniffs flight hole directly
Passing mound	Point event	When an individual is seen in frame but walks by the mound without stopping to engage in any inspection behaviors or termite-fish
Successful dip	Point event	When an individual dips a tool into the mound and successfully extracts and consumes termites confirmed by seeing them chew but not for tool modification
Tactile inspection	Point event	When an individual removes small qualities of substrate from the mound’s surface with their hand or foot by scratching at one spot on the mound with their fingers or toes
Unsuccessful dip	Point event	Each time a tool is inserted into the mound but the individual does not subsequently insert the tool into their mouth and chew
Visual inspection	Point event	When the individual stops traveling and their gaze is either staring directly at one portion of the mound or scanning the surface

State events are behaviors that record the duration of a behavior while point events do not record duration. Inspection behaviors include olfactory inspection, tactile inspection, and visual inspection.

We analyzed data in R version 3.4.4^35^. Previous research on termite ecology at Issa showed that cumulative rainfall and weekly rainfall had an effect on seasonal termite activity patterns^15^. Additionally, wind speed and cumulative rainfall were the two most significant factors influencing alate dispersal at this site. For this study, we deliberately did not test for all possible weather parameters at our disposal so as to avoid randomly stumbling on to significant *p*-values. Instead, given the results of Phillips and colleagues recent study^15^, we decided to focus on rainfall parameters for our analysis of chimpanzee termite foraging behavior. The rainfall associated variable “weekly rainfall” is defined by a rolling average value of the daily precipitation from the seven days preceding a given observation, whereas “cumulative rainfall” is the total accumulated precipitation since the onset of the most recent dry season and the day of the observation. We checked for Pearson’s correlation coefficient between these two variables to ensure that they did not correlate. We identified the onset of annual dry seasons as the first period of 30 or more days without daily aggregate rainfall ≥ 0.3 mm^15^.

We ran six regression models (see below) to test our hypotheses and better understand the relationship between precipitation and chimpanzee arrival with prepared tools, inspection behavior, and termite-fishing. All models were mixed-effect models including a random nested parameter of an individual subject within an individual mound. Three of our models were logistic regressions prompting us to use generalized linear mixed-effect models (GLMM). We utilized linear mixed-effect models (LMM) for the three non-logistic models.

All models include the same independent fixed effects of weekly average rainfall, cumulative rainfall, and the random fixed effect of individual within a mound. We additionally included a quadratic term for cumulative rainfall, predicting that chimpanzee behaviors would similarly increase in frequency or duration as rain initially began to accumulate but decline as rainfall became heavy and more consistent in the late wet season. All of the rainfall covariates were zero-transformed. We considered the possibility that chimpanzees may incorporate both very recent rainfall trends (weekly average rainfall) as well as broader seasonal rainfall (cumulative rainfall) at the same time when making decisions about visiting termite mounds. Thus, for each of our models we used ANOVA to compare the fit of model in which the rainfall parameters interact versus a model in which the rainfall parameters do not interact to determine whether the interactive model significantly outperformed the non-interactive model.

We examined the variance inflation factor (VIF) values for models with two or more fixed effects and always found values under 1.5 (Table 3) suggesting collinearity was not an issue. For each model with a gaussian distribution, we examined their histogram of residuals, qq plots, as well as the residual values against fitted values. No resulting plots violated normality assumptions. Additionally, the response variables for all models with gaussian distributions were log transformed to reduce skewness. For every model, we ran an analysis of variance test (ANOVA) to compare our explanatory model with a respective null model which excluded all fixed effects and included only the random slope term of variation by chimpanzee at a specific mound.

**Table 3:** List of mixed-effect model formulas. The abbreviation avg rain = average weekly rainfall and cml rain = Cumulative rainfall.

Model	model formulation	Distribution	Interactive terms	VIFs
1	Arrive with tool ~ avg rain * (cml rain + I(cml. rain^2^)) + (1|mound / subject)	Binomial	Yes (*p* = 0.012)	< 1.3
2A	Inspect binomial ~ avg rain * (cml rain + I(cml rain^2^)) + (1|mound / subject)	Binomial	Yes (*p* < 0.001)	< 1.3
2B	Inspect duration ~ avg rain + (cml rain + I(cml rain^2^)) + (1|mound / subject)	Gaussian	No (*p* = 0.28)	< 1.2
3A	Fishing binomial ~ avg rain * (cml rain + I(cml rain^2^)) + (1|mound / subject)	Binomial	Yes (*p* < 0.001)	< 1.5
3B	Fishing duration ~ avg rain + (cml rain + I(cml rain^2^)) + (1|mound / subject)	Gaussian	No (*p* = 0.808)	< 1.2
3C	Fishing efficiency ~ avg rain + (cml rain + I(cml rain^2^)) + (1|mound / subject)	Gaussian	No (*p* = 0.181)	< 1.2

I() is a function in R that keeps operators within term (in this case ^2) as regular operators instead of special formula operators. ANOVA tests used to compare the fit of interactive versus non-interactive models with rainfall covariates.

All data presented in this study were obtained via non-invasive monitoring (motion triggered cameras) of a wild animal population in full accordance with and under permission from the Tanzanian wildlife research authorities under research permit number 2017–366-NA-2017–341 (Tanzania Commission for Science and Technology). As such, no experiments on live animals were conducted and hence the ARRIVE 2.0 guidelines do not apply to this study.

## Results

**Fig. 1:**
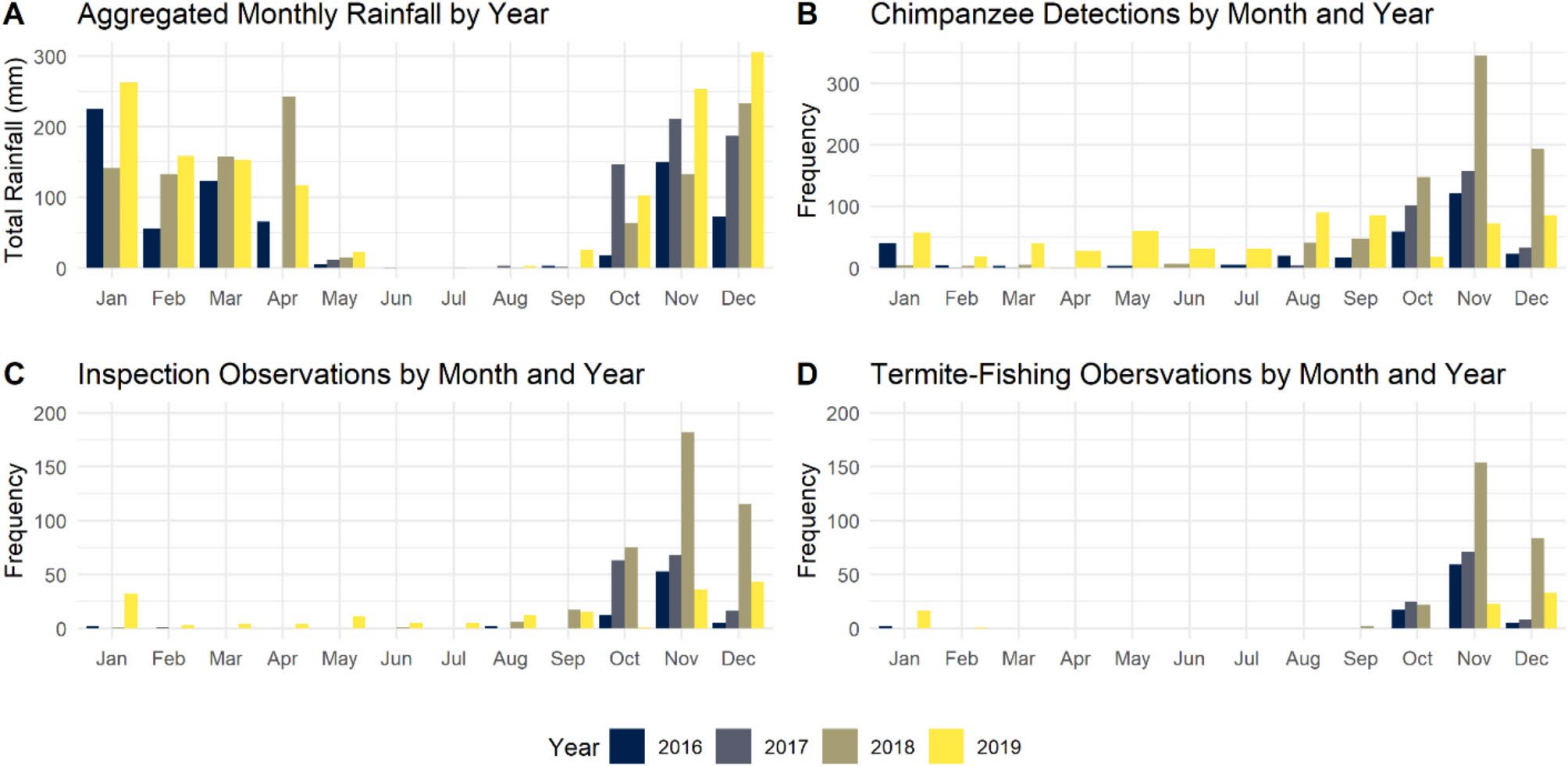
(**A**) Histogram of aggregated monthly rainfall by year. Note the gap in rainfall data between January and April of 2017 due to equipment malfunction, but general trends can be expected to be comparable to the other three years. (**B**) Detections of chimpanzees detected by camera traps set up at or near termite mounds by month and year. (**C**) Chimpanzee inspection behaviors at termite mounds by month and year. (**D**) Observations of chimpanzees successfully termite-fishing by month and year.

We analyzed 2,006 observations of fully weaned chimpanzees at termite mounds over the four-year period 2016–2019. From these observations, we recorded a total of 165.6 h of chimpanzee behavior at termite mounds. Note this number is higher than 113 h of footage with chimpanzees reported earlier because, in many instances, more than one chimpanzee’s behavior was recorded simultaneously at a mound. Four out of the six mixed-effect models suggest either weekly rainfall, cumulative rainfall, or a combination of both is associated with the probability that chimpanzees inspect a mound or succeed in extracting termite prey. Figure 1 displays frequency distributions for chimpanzee detections, inspection observations, and termite-fishing observations by month and year alongside a histogram of monthly aggregated rainfall data for each year during our study period.

### Chimpanzee arrival at mound with a tool

**Fig. 2:**
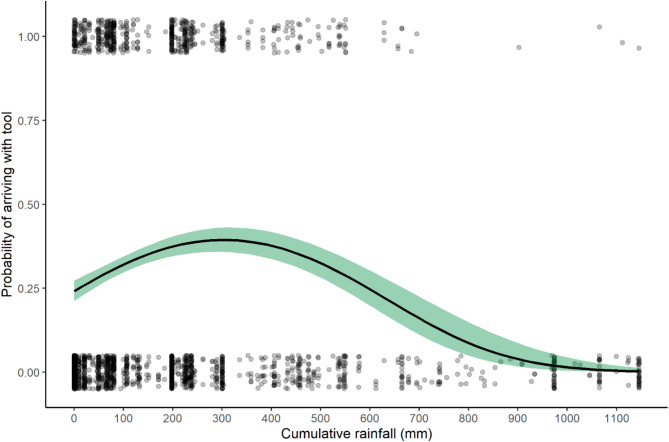
The effect of cumulative rainfall on chimpanzees arriving to termite mounds with a termite-fishing tool. Shaded band represents 95% confidence interval. Points are jittered in order to view number of observations. The probability that a chimpanzee arrived to a mound with a prepared tool rises immediately after the first seasonal rains and declines dramatically in the late wet season.

Model 1 was significant compared to the null model (*p* < 0.001), driven by the effects of cumulative rainfall (*p* < 0.001) (Fig. 2) and the interaction between cumulative rainfall and weekly average rainfall (*p* = 0.003) (see Table S1 for details). There were 592 instances in which a chimpanzee arrived at a termite mound with a termite-fishing tool. In 323 of these instances, the chimpanzee arrived at a mound with a tool, but was not able to successfully termite-fish.

### Chimpanzee inspection behavior

**Fig. 3:**
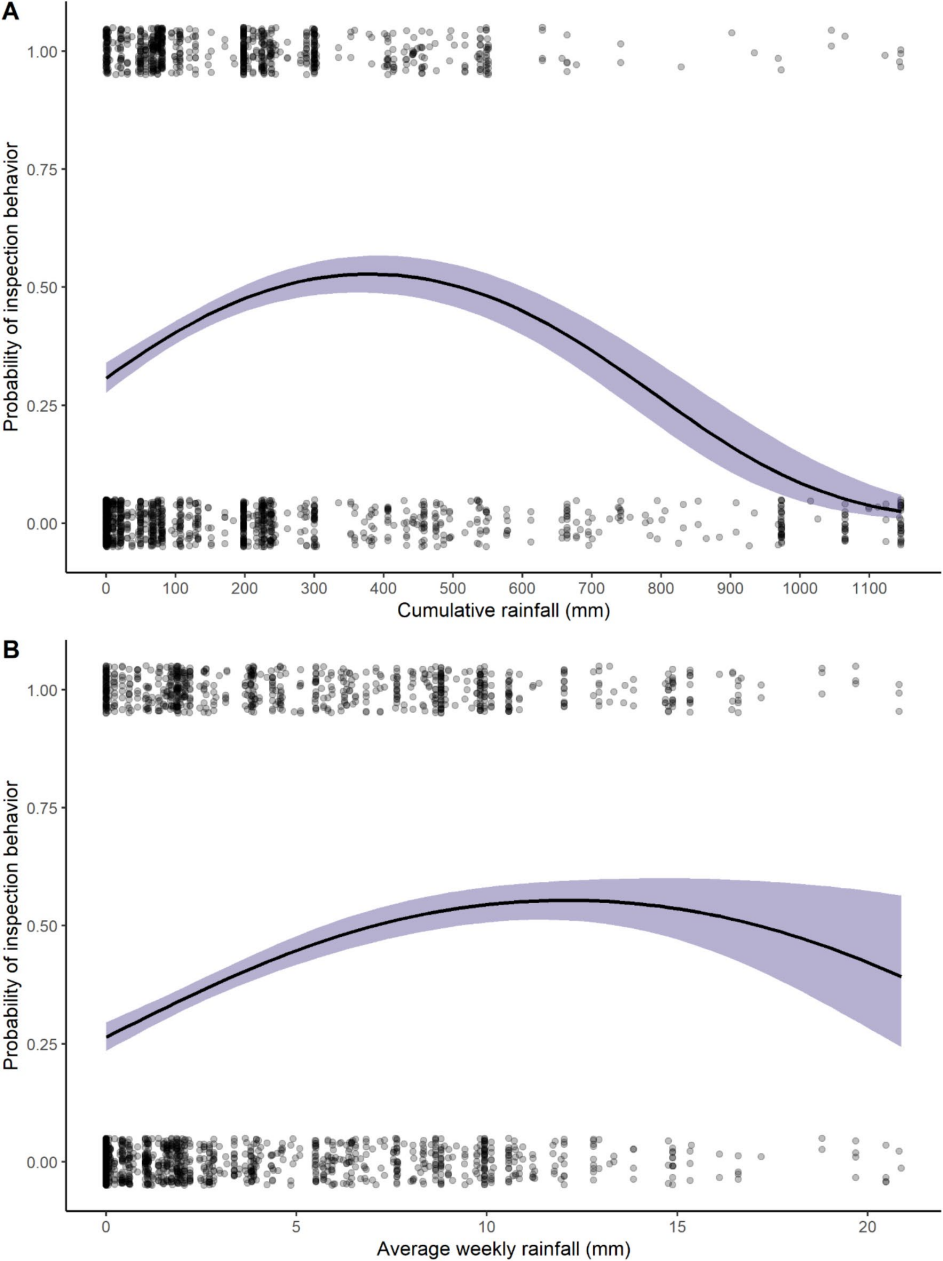
(**A**) The effect of cumulative rainfall on the binary outcome of whether a chimpanzee inspected a termite mound for termite-fishing viability. (**B**) The effect of average weekly rainfall on whether a chimpanzee inspected a termite mound for termite-fishing viability. Shaded bands represent 95% confidence intervals. Points are jittered in order to view number of observations. Interaction between rainfall terms has a significant effect on probability of inspection behavior. Probability of inspection increases with initial seasonal rains and increased average weekly rainfall but then decreases as rainfall continues to accumulate and the weekly average rainfall increases. Inspection probability decreases into the late wet season and there is less precision in probability estimates at the highest range of average weekly rainfall.

Model 2A performed significantly better than the null model (*p* < 0.001). Cumulative rainfall (*p* < 0.001) (Fig. 3A), weekly rainfall (*p* = 0.002) (Fig. 3B) as well as the interaction between weekly rainfall and cumulative rainfall values (*p* < 0.001) were significant predictors for the occurrence of a chimpanzee inspection behavior (See Table S1 for details). There were 789 instances in which we identified an individual to engage in a specific inspection behavior (olfactory, tactile, or visual). In 328 of these instances successful extraction of termites occurred and in 461 instances the individual left the mound before they were observed to successfully termite-fish. In some cases, a bout would begin with chimpanzees already successfully extracting termites before inspection behavior could be observed.

**Fig. 4:**
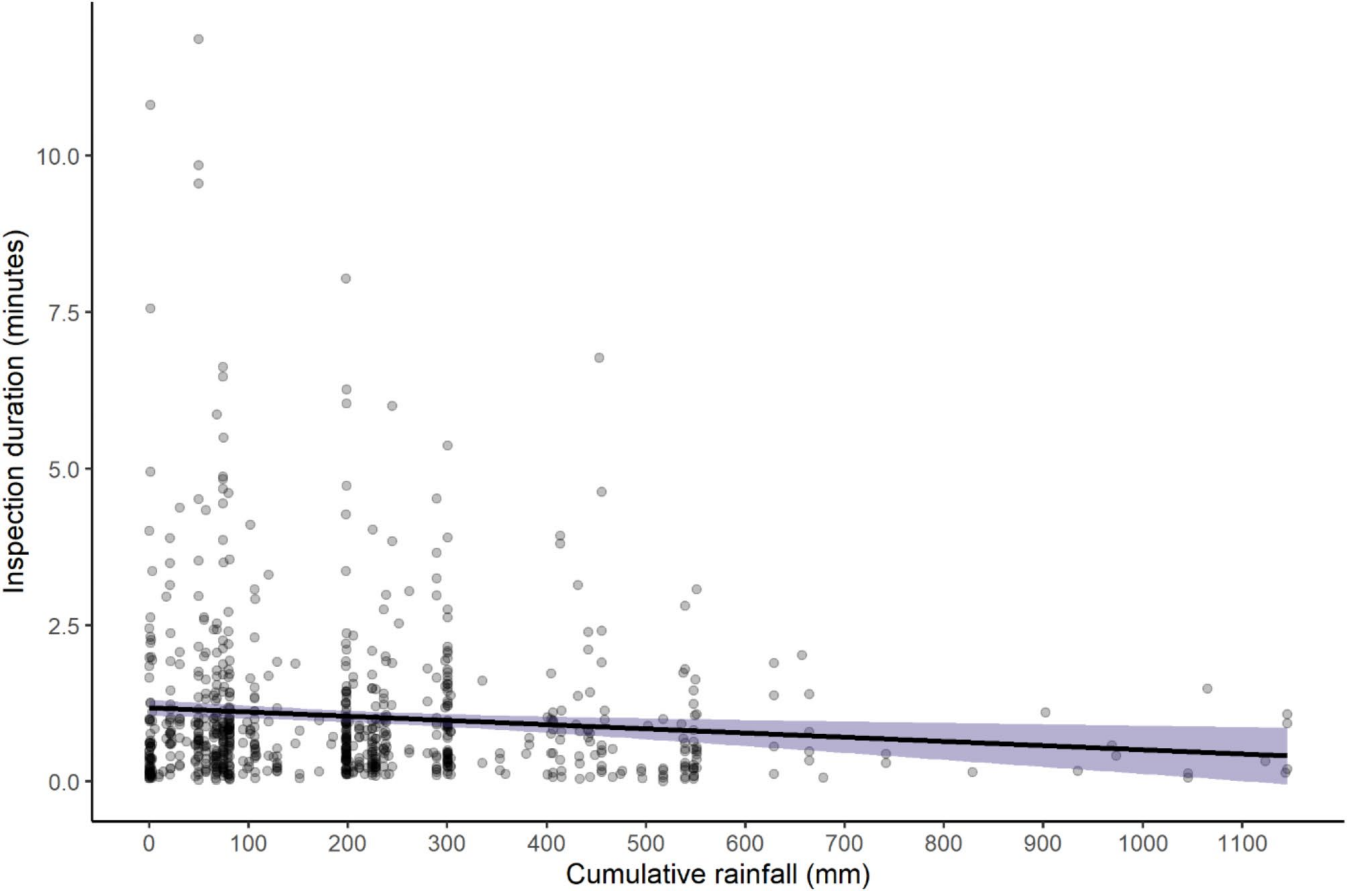
The effect of cumulative rainfall on the duration of inspection behavior at termite mounds. The shaded band represents 95% confidence interval. The duration of inspections steadily decline as rainfall accumulates into the late wet season.

Model 2B performed better than the null model (*p* = 0.015), driven by the effect of cumulative rainfall (*p* = 0.044) (Fig. 4) on the duration of inspection behaviors (See Table S1 for details). The average length of time that a chimpanzee would inspect a termite mound was 62 s with a standard deviation of 78 s. The maximum amount of time a chimpanzee spent inspecting a termite mound was 11.9 min.

### Chimpanzee termite-fishing behavior

**Fig. 5:**
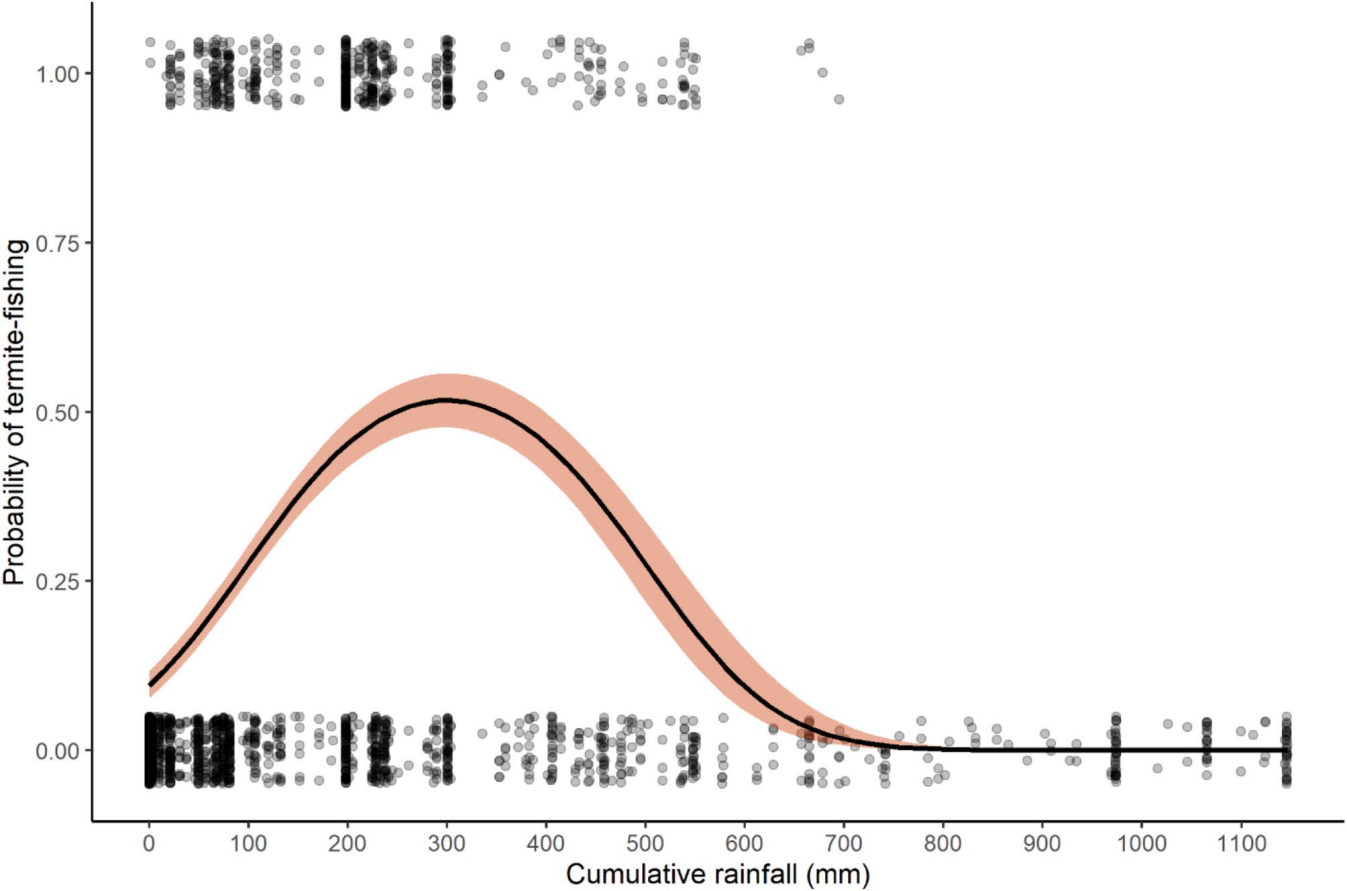
The effect of cumulative rainfall on the binary outcome of whether a chimpanzee successfully extracted termites from a termite mound. The shaded band represents 95% confidence interval. Points are jittered in order to observe number of observations. The probability for a successful termite-fishing bout to occur begins to rise after first rains of the wet season and peaks at around 300 mm of cumulative rainfall.

Model 3A performed significantly better than the null model (*p* < 0.001), which was driven by the significant effects of cumulative rainfall (*p* < 0.001) (Fig. 5) and the interaction between cumulative rainfall and weekly average rainfall (*p* = 0.002) (see Table S1 for details). There were 522 instances in which an individual chimpanzee had a successful termite-fishing bout.

Model 3B did not perform significantly better than the null model suggesting no effect of rainfall on the duration of termite-fishing bouts. Model 3C did not perform significantly better than the null model suggesting no effect of rainfall on the amount of successful extraction of termites within a termite-fishing bout.

## Discussion

The combined results of the present study and its precursor on Issa *Macrotermes* seasonality^15^ suggests how seasonal variation in *Macrotermes* surface activity is an obstacle that chimpanzees may navigate by utilizing rainfall cues. During the late wet season and throughout the dry season, *Macrotermes* are rarely accessible at the surface of their mounds^15^. During that time, Issa chimpanzees rarely prepare tools or inspect termite mounds (Figs. 1, 2, 3A). During that time, we regularly observe chimpanzees bypassing mounds without attempting to inspect them. These non-events^21^ suggest that Issa chimpanzees understand that *Macrotermes* prey are not accessible during late wet season or dry season conditions and do not invest time and energy into exploring this food resource (Fig. 3A,B).

Once the rains begin, Issa chimpanzees begin to make tools, inspect termite mounds increasingly frequently, and have preliminary success in extracting termites. That there is a steep rise in inspection rates and arrivals with tools at the first onset of rains (Figs. 1C, 2, 3A), but a less steep rise in successful termite-fishing bouts (Figs. 1D and 5) suggests that chimpanzees anticipate their ability to find termites, potentially based on the onset of rainfall after the dry season, even if they are not quite yet accessible at a given mound. This can be considered an instance of “informative failing”^21^ as the inspection behavior yet inability to find embedded foods provides information on the chimpanzees’ expectations. These failures to termite-fish are different from the non-events prevalent in the late wet season and dry season when chimpanzees bypass mounds without stopping to inspect or attempt to termite-fish. Similar spikes in termite-fishing intensity at the onset of wet seasons are reported at Fongoli^13^ and Gombe^26^. However, both sites appear to have more consistent foraging of the resource throughout the year compared to the Issa Valley results presented here.

As rainfall further accumulates in the 50-100 mm range, *Macrotermes* become more accessible at the surface of their mounds^15^ and we observed an increase in the proportion of successful termite-fishing bouts (Fig. 5). Direct observations suggest that termite-fishing behavior begins at almost the same time as the onset of *Macrotermes* accessibility, rather than being related to a reduction in preferred plant food availability or hunting of mammalian protein^20^. These findings show clearly that Issa chimpanzee tool use is driven by the opportunity to gather a high value, ephemeral resource (termites) rather than the necessity to rely on fallback foods during a period of resource scarcity^4,36^.

Converging analyses on the evolution of primate intelligence make the case that reliance on high-value, but time-costly resources better explain, and are more correlated to, increased cognitive capacities in primates rather than intricate social systems^27,37^. The utilization of specific weather cues to predict and forage for high value resources is a cognitive ability that has been previously identified in wild primate populations. Janmaat and colleagues^38^ found that mangabeys (*Lophocebus albigena johnstonii*) in the Kibale Forest of Uganda are more likely to revisit fig trees (*Ficus* sp*.*) on days with higher average maximum temperatures and solar radiation. These conditions are known to influence the maturation of figs and thus the researchers concluded mangabeys are able to efficiently predict fig availability based on weather cues. Our study shows that Issa chimpanzees appear to similarly benefit from the ability to adjust their tool construction and inspection effort at termite mounds depending on rainfall conditions. This conclusion identifies the temporality of not only plant foods but also eusocial insects as a possible selective pressure contributing to cognitive adaptation as well as tool use in primates^39^.

Besides these cognitive skills, the analysis of hierarchical organization and sequential behavior necessary for effective tool use has helped to illuminate cognitive abilities in previous studies^40^. Corrective guidance is a cognitive skill that refers to the ability of an individual to adjust a behavioral sequence and correct their anticipatory schema in response to cues by pausing, skipping, abandoning, proceeding, and correcting errors in sequential steps^6,8^. Close analysis of inspection behavior routines at termite mounds were outside the scope of this study. Yet this may be a fruitful avenue for future study based on the results of model 2B in which we observe a difference in the duration of inspection sequences (Fig. 4). Chimpanzees are likely adjusting inspection sequences as a form of corrective guidance depending on various mound conditions and the outcome of previous inspections tests (e.g. sniffing tools or scratching soil, see supplementary video). Future research may illuminate the structure of these routines and whether behavioral sequences vary significantly between individuals, sexes, or communities.

## Conclusions

Issa chimpanzees increase their tool preparation and mound inspection efforts after the onset of rains following the extended dry season. Issa chimpanzees are rewarded with a higher probability to successfully termite-fish by preferentially foraging for termites during the early wet season. Our study demonstrates that primate foraging cognition is not only useful for feeding on ephemeral and nutrient dense plant foods, but also plays and important role in predation on eusocial insects. We suggest that this ability may likely extend to other forms of foraging and species-interactions which could be explored in the future, such as hunting immature monkeys^41,42^ or foraging for macrofungi^43^. In addition to tool-use^39^, the utilization of weather cues may be a cognitive adaptation allowing for the exploitation of seasonally embedded, high-value food resources. Identifying these resources limited in time and/or space as well as the patterns of cognition among the primates that forage for them are important aspects for evaluating hypotheses on the evolution of intelligence in primates and other non-human animals^21,27^.

Termite-fishing behaviors are also of notable interest to hominin foraging patterns since their first potential linkage to *Australopithecus robustus*^44^. Stable carbon isotopic signatures among South African hominins have led to speculation that termites may have played an important dietary role for hominin populations^45,46^, though these C_4_ signatures are not necessarily derived from termites^2,32,47,48^. Early hominin populations certainly had access to termite mounds and may have utilized various tools, like chimpanzees, to access them^49^. Our results suggest that hominins would have had rainfall as a predictive cue for successful termite foraging. This nutrient-dense and low-risk resource would have been seasonally predictable and readily available as a dietary supplement, potentially for multiple species of Plio-Pleistocene hominins. And though wood tool use is a perishable technology, the multi-step and planned technological use reported here for Issa chimpanzees fits well within what Pascual-Garrido and colleagues^50^ call a “perishable-to-lithic behavioral continuum” (pg.3). Documenting plant technological use and quantifying the circumstances in which these technologies are used is a primary and necessary step toward both understanding the nuanced foraging behaviors of extant chimpanzees and toward developing testable hypotheses for past hominin behaviors.

## Electronic supplementary material

Below is the link to the electronic supplementary material.

Supplementary Material 1

Supplementary Material 2

Supplementary Material 3

## Data Availability

Data used for analysis is available in the article or supplementary materials. For access to the raw video footage, please contact VO and footage can be made available upon reasonable request.
